# Percutaneous cholecystostomy in acute cholecystitis; a retrospective analysis of a large series of 104 patients

**DOI:** 10.1186/s12893-015-0002-8

**Published:** 2015-03-08

**Authors:** Asgaut Viste, Dag Jensen, Jon Helge Angelsen, Dag Hoem

**Affiliations:** Department of Acute and Gastrointestinal surgery, Haukeland University Hospital, N-5021 Bergen, Norway; Department of Radiology, Haukeland University Hospital, Bergen, Norway; Department of Clinical Medicine K1, University of Bergen, Bergen, Norway

**Keywords:** Cholecystitis, Cholecystostomy, Percutaneous, Complications

## Abstract

**Background:**

The purpose of this study was to evaluate the clinical course and possible benefit of a percutaneous cholecystostomy in patients with acute cholecystitis.

**Methods:**

Retrospective study of 104 patients with severe cholecystitis or cholecystitis not responding to antibiotic therapy treated with percutaneous drainage of the gall bladder (PC) during the period 2007 – 2013. Primary outcome was relief of cholecystitis, complications following the procedure and need for later cholecystectomy.

**Results:**

There were 57 men and 47 women with a median age of 73,5 years (range 22 – 96). 43% of the patients were ASA III or IV and 91% had cholecystitis Grade 2 or 3. About 60% of the patients had severe comorbidity (cardiovascular disease or active cancer). Drain insertion was successful in all but one patient and complications were mild, apart from two patients that needed percutaneous drainage of intraabdominal fluid collection due to bile leakage. The drain was left in place for 1 – 75 days (median 6,5). When evaluated clinically and by blood tests (CRP and white blood cell counts) we found resolution of symptoms in 101 patients (97,2%), whereas 2 patients had no obvious effect of drainage. Four patients died within 30 days, no deaths were related to the drainage procedure. Follow-up after drainage was median 12 months (range 0 – 78). During that time cholecystectomy was performed in 30 patients and 24 patients had died. Following cholecystectomy, two had died, both from cancer and more than one year after the operation.

**Conclusion:**

Patients with acute cholecystitis were promptly relieved from their symptoms following PC. There were only minor complications following the procedure and only about 30% of the patients had a later cholecystectomy.

## Background

Acute cholecystitis is a commonly encountered disease in surgical departments and the number of treated patients is increasing as the population ages. Standard treatment for acute cholecystitis has for many years been laparoscopic cholecystectomy (LC) in the acute phase. Many of these patients are, however, multi-morbid and not fit for surgery, whereas laparoscopic cholecystectomy is the treatment of choice in young, fit patients. LC might be a relatively simple procedure when undertaken during the early phase of the disease, whereas LC during the period one to six weeks after onset of illness by most authors is considered more challenging and in some cases even hazardous [1-4]. Lee et al. has, however, documented that there is no difference whether the operation was performed as an emergency, in the intermediate interval or after five weeks [5]. The scientific evidence for advocating any specific therapy for acute calculous cholecystitis is sparse. Even the Tokyo guidelines for acute cholecystitis are mainly based on opinions among well-known surgeons and not on high-quality scientific evidence [6]. Some researchers have also questioned the necessity at all for surgery in acute cholecystitis, even in the long term [7,8]. Conservative treatment with antibiotics and percutaneous drainage are reported as an adequate alternative to surgery, although most authors recommend delayed cholecystectomy in these cases [9,10].

The purpose of this study was to evaluate the efficacy of percutaneous drainage of the gall bladder in patients with severe acute cholecystitis or cholecystitis not responding to antibiotic therapy. Main outcome was resolution of cholecystitis, postoperative complications and later need for cholecystectomy.

## Methods

### Settings

Haukeland university hospital is a tertiary referral hospital as well as a local hospital for emergency cases for 350.000 patients in the Bergen area in Western Norway. The hospital’s standard treatment for acute cholecystitis is antibiotics for the first days with continuous monitoring of the effect. Cholecystectomy is principally an option in the acute phase in young and fit patients, but is normally not performed during night time. In frail patients with severe cholecystitis and comorbidity percutaneous cholecystostomy (PC) is performed at an early stage. In other patients PC is performed if the condition is worsening or not improving. Diagnosis was confirmed with ultrasonography or CT-scan as found appropriate.

### Patients

Data for all consecutive patients undergoing (PC) were retrieved from the database at the department of radiology. Patients having PC for other indications than acute cholecystitis were excluded from analyses. The diagnosis of acute cholecystitis was based on a combination of findings at clinical examination (right upper quadrant tenderness and Murphy sign), laboratory data (leukocytosis, CRP), and sonographic evidence of gallstones, thickened gallbladder wall, pericholecystic fluid, and/or sonographic Murphy’s sign. Patients were stratified as mild, moderate and severe AC according to the Tokyo classification [11]. Percutaneous drainage of the gall bladder was performed by a specialized intervention radiologist under conscious sedation and local anesthetics. The applied procedure was either Seldinger’s technique or a direct puncture of the gall bladder guided by ultrasonography, preferably through a small brim of the liver. A 7 or 8 Fr catheter was introduced into the gall bladder and secured in place, and the position confirmed by fluoroscopy. Some days later a fluoroscopic study was performed in order to evaluate if the cystic duct was open and if there was a free passage to the duodenum. At that time the drain could be removed. If no passage through the cystic duct was confirmed by cholangiography, the drain was left in place and the patient discharged. Another fluoroscopic study was performed about two weeks later and the drain was then removed. Patients’ outcome was evaluated clinically as well as by laboratory tests like CRP and blood leucocytes. No patients with acute cholecystitis were offered cholecystectomy in the acute phase. For a few young patients an appointment was made for laparoscopic cholecystectomy a few months later, whereas most patients were observed for further symptoms.

Data analyses were performed by IBM statistics SPSS version 21 and analyzed by frequency tables, crosstabs and box plots for continuous variables. Variables not normally distributed were analyzed by non-parametric tests.

Since this study was defined as a quality assurance project and not a prospective study it is exempted from review by the Regional Committee for Medical and Health Research Ethics, Western Norway.

## Results

During the period 2007 – October 2013 a total of 104 patients had a percutaneous cholecystostomy (PC) due to suspected acute cholecystitis. There were 57 men and 47 women with a median age of 73,5 years (range 22 – 96).

Twenty-four patients had a history of previous bile colic and 15 had undergone a previous episode of acute cholecystitis. Nineteen patients (18,4%) had a perforation of the gall bladder as evaluated by CT or ultrasonography before PC. Insertion of the drain was performed median two days after admittance to the hospital (range 0 – 22 days). Final diagnosis following drainage concluded with acalculous cholecystitis in 18 and calculous cholecystitis in 86 patients (Table 1). Seventy-six percent of the patients had a blocked cystic duct as evaluated by the contrast study. Macroscopic drainage of pus was reported in 17 patients with E-coli as the most commonly cultured bacteria in 20 patients (Table 2). Clinical and outcome parameters were also analysed by patient age. Apart from more comorbidities and cases with cancer there were no significant differences between the Groups above or below median age of 73 (Table 3).

**Table 1:** **Patient and disease characteristics for 104 patients treated with percutaneous gall bladder drainage**

**Variable**	**Patients**	**%**
Age (median, range)	73,5	22 - 96
Sex		
Men	57	54,8
Women	47	45,2
Comorbidity		
Cardio-pulmonary	48	46,2
Cancer	13	12,5
Other	13	12,5
None	28	26,9
Not stated	2	1,9
Diagnosis		
Calculous cholecystitis	86	82,7
Acalculous cholecystitis	18	17,3
ASA score		
I	16	15,4
II	44	42,3
III	39	37,5
IV	5	4,8
Tokyo classification		
Grade 1 (mild)	10	9,6
Grade 2 (moderate)	87	83,7
Grade 3 (severe)	7	6,7
Previous bile colic		
Yes	24	23,1
No	80	76,9
Previous cholecystitis		
Yes	15	14,4
No	89	85,6
White blood cell counts (median, range)		
Day 0	14,0	1,8 – 35,0
Day 2	9,6	2,2 – 33,0
Day 4	8,8	1,9 – 35,0

**Table 2:** **Results following percutaneous cholecystostomy in 104 patients with acute cholecystitis**

	**No.**	**%**
Contrast passage through cystic duct at time of drain insertion		
Into common bile duct	10	9,6
Into duodenum	4	3,8
No passage	79	76,0
Obstructed cystic duct	11	10,6
Bacteriology		
Growth (unspecified)	10	9,6
E-coli	17	16,3
Enterococcus	8	7,7
Anaerobes	1	1,0
Fungus	1	1,0
E-coli + enterococcus	3	2,9
Others	7	6,7
No growth	15	14,4
Not obtained	43	41,3
Complications		
Drain dislocation	7	7,6
Leakage	2	1,9
Obstructed drain	1	1,0
Unsuccessful	1	1,0
No information	2	1,9
Symptoms at follow-up		
Gall bladder/bile duct related	28	26,9
No symptoms	54	51,9
Not stated	22	21,7
Later operations		
Cholecystectomy		
Laparoscopic	19	18,3
Open (planned)	3	2,9
Converted to open	8	7,7
Sphincterotomy	8	7,7
PTD	2	1,9
No operation	62	59,6
Not stated	2	1,9

**Table 3:** **Clinical and outcome parameters versus age group for patients with PC (median age = 73)**

	**Age <73**	**Age >74**
**Variable**	**N**	**N**
Sex M/F	26/25	31/22
ASA		
I	16	0
II	21	23
III	13	26
IV	1	4
Comorbidity		
No	24	4
Cardio-pulmonary	16	32
Cancer	3	10
Other	8	5
Tokyo classification		
Mild	6	4
Moderate	40	47
Severe	5	2
Gall bladder stones		
Yes	41	43
No	9	10
Complications to PC		
Dislocation	4	3
No complications	44	47
Days with drain	6,5	6,5
Length of stay postdrain (median)	9	10
Later cholecystectomy		
Yes	29	2
No	20	43
Sphincterotomy	1	7

Insertion of the drain was successful in all but one patient, in which the gall bladder was completely impacted with stones. Three patients had repositioning or reinsertion of their drains. Seven drains were found to be dislocated with part of it being located intraabdominally at control fluoroscopy. One drain was occluded and two patients had a bile leak from the site of insertion in the gall bladder and into the abdomen. These two patients were successfully treated with percutaneous drainage. Two drains were changed due to technical problems (occluded drain and extraabdominal leak). One patient had a PTD in addition to the gall bladder drain due to continuous septicemia (Table 2). After insertion of the drain some patients complained of abdominal pain. This was thought to be related to minor bile leakage and pain was easily relieved by analgesics. Apart from this there were no serious complications reported. Drains were removed median 6,5 days after insertion (range 1 – 75 days).

**Figure 1:**
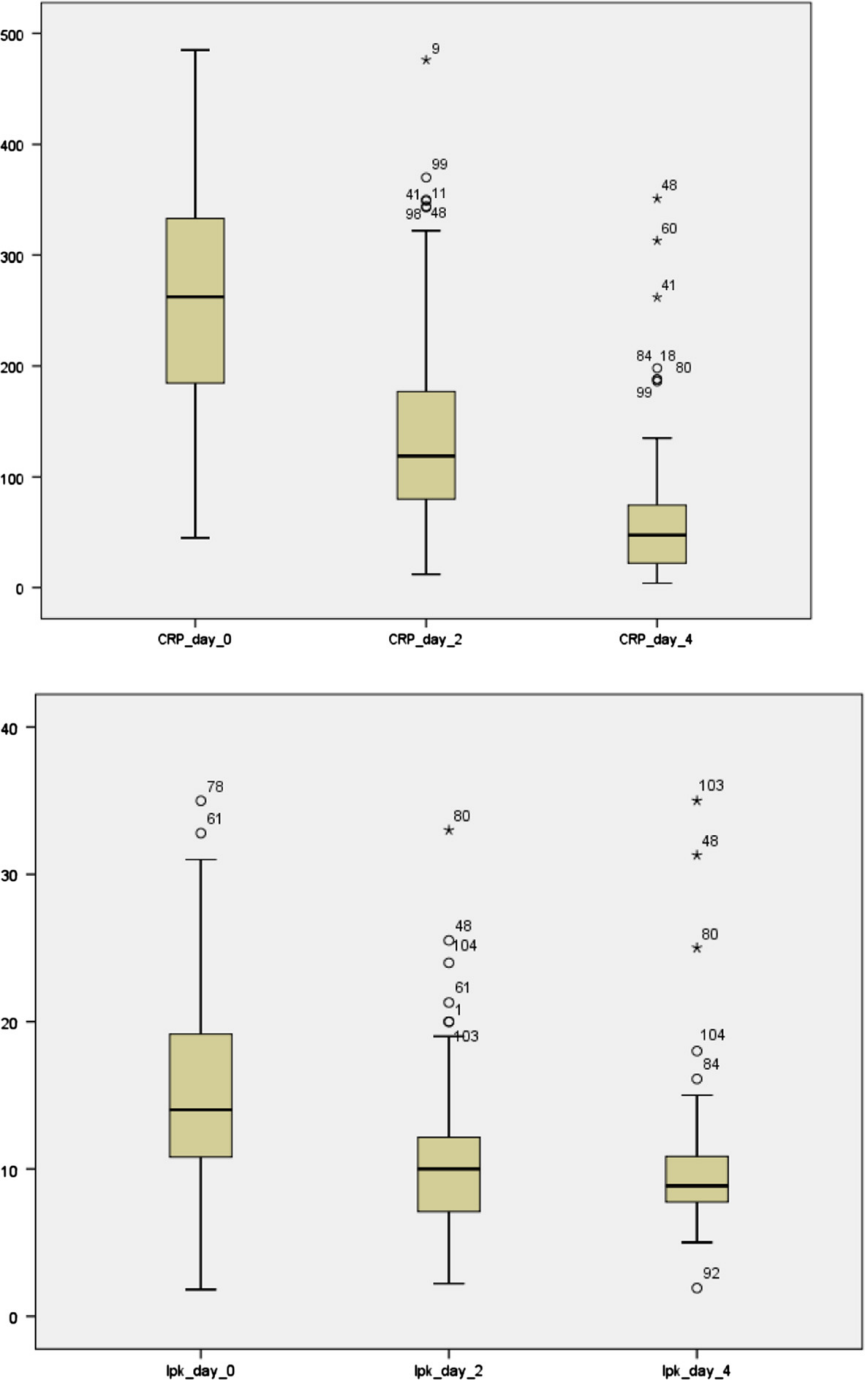
**CRP (101 patients) and WBC (99 patients) at day of drain insertion (day 0), day 2 and day 4 in patients treated with percutaneous cholecystostomy for acute cholecystitis.**

In addition to the clinical evaluation, effect of drainage was also evaluated by CRP and white blood cell count (WBC). CRP at the day of drainage was median 263 (range 32 – 485), at day two 124 (range 12 – 476) and at day four 48 (range 4 – 351) (Figure 1). CRP and WBC were not normally distributed and Wilcoxon’s nonparametric test revealed a significant reduction from day 0 to day 2 and day 4 for both variables. Resolution of symptoms occurred in 101 patients (97%), whereas in two patients there was no obvious effect following drainage (not stated in one patient).

Postoperative mortality within 30 days was 4 of 105 patients (3,8%). Two of these were related to their biliary disease and none to the drain. One 81 year old patient with a cardiovascular history (ASA 2) was found dead in his bed three days after drainage, whereas the other patient was a 91 year old woman that died the day after drainage with a cardiovascular history (ASA 4) and bile sepsis. Median length of stay post-drainage was seven days (range 1 – 53). Total hospital stay was median 10 days (range 1–53).

Follow-up after drainage was median 12 months (range 0 – 78). During that time 25 patients had died. No patients having undergone laparoscopic cholecystectomy had died, whereas two patients had died following open cholecystectomy, both from cancer and more than one year postoperatively.

## Discussion

The main findings in this study were that gall bladder drainage in most patients had an immediate and beneficial effect on acute cholecystitis. Furthermore, the procedure could be performed without almost any major complications.

According to the Tokyo guidelines mild (Grade I) acute cholecystitis in an early stage should be treated with cholecystectomy, preferably laparoscopic, whereas moderate and severe (Grade II and III) cholecystitis lasting longer than 5 – 7 days should be treated conservatively with antibiotics and eventually also percutaneous cholecystostomy, followed by a delayed cholecystectomy [11]. These recommendations are, however, not supported by high-quality scientific evidence, but developed based on best clinical evidence and discussions at the international Consensus Meeting held in Tokyo on April 1–2, 2006. Some studies have questioned this statement and even shown that the results are equivalent or even better if cholecystectomy is performed early [1,2,12], or even in the intermediate period one to five weeks after onset. A few small studies have compared cholecystectomy with antibiotic therapy and the conclusion of these are that about one third of the conservatively treated patients will need a cholecystectomy later due to new symptoms [7,8]. This is in keeping with what we found where 30% had a later cholecystectomy. It should, however, be noted that a high proportion of these patients needed a conversion from laparoscopic to open surgery due to severe intraoperative adhesions. Furthermore, it is recommended that patients with a severe cholecystitis and patients with extensive comorbidity might benefit from percutaneous drainage of the gall bladder and with cholecystectomy later on [6]. Our main treatment policy for acute cholecystitis has been antibiotic therapy in the early phase. When the response to this is not satisfactory the patients are offered percutaneous drainage of the gall bladder. During the later years the indications for such drainage have been more liberal due to good results and minor complications following the procedure. For this reason we have also drained some rather young patients with minor comorbidities when the response to antibiotic therapy was not satisfactory. It should, however, be noted that about 50% of our patients had cardiovascular diseases or cancer, and that about the same proportion were classified as ASA III and IV. When this treatment strategy is successful the patients are evaluated as out-patients for a later cholecystectomy if they still have attacks of biliary colic. In our setting cholecystectomy is not a mandatory procedure after cholecystitis. The rationale for this policy is supported by several studies which have documented that about one third of the patients will need a later cholecystectomy [7,8].

We found that PC could be performed with a low complication rate. The outcome was also satisfactory with a symptomatic relief in 96% of the patients and a low rate of complications. This is in accordance with the findings of Byrne et al. [13] that documented a complication rate of 4,5% and a 100% success rate. Although the cystic duct most often was blocked at the time of drain insertion, this blockage was in most cases resolved during the control study some days later. It should also be noted that the drain could be removed safely without any severe leakage. There is an ongoing debate in the literature if this might be related to the technique of drain insertion whereby the drain is introduced through a small brim of the liver [10,13]. The main reason for the long median time of draining was related to some patients being discharged with their drains in place and removed at a later stage at a visit to the out-patient clinic.

30-day post-procedure mortality was in this series 3,6%. This is of the same order as what was reported in the review paper of Winbladh et al. [14] for acute cholecystectomy (4%) and far lower than they reported for PC (15,4%).

So far, there are only two published randomized studies related to percutaneous drainage of the gallbladder in calculous cholecystitis. Hatzidakis et al. found no difference in complications following PC and antibiotics versus conservative treatment only [15], whereas Akyurek et al. concluded that PC and early laparoscopic cholecystectomy (LC) was favorable compared to PC and LC later on [16]. In a review including those randomized trials Gurusamy et al. concluded that so far there is no hard evidence concerning the benefit of PC in acute cholecystitis [17]. This was also the conclusion in the systematic review paper of PC [13]. The results following the Dutch “Chocolate study” randomizing between PC and LC in severely morbid patients will hopefully solve some of these issues [18]. It might be questioned if a pragmatic use of PC in severe acute cholecystitis represents a cost-effective strategy. Obviously, most studies document that the hospital stay is shorter when laparoscopic cholecystectomy is performed early versus later on [19,20]. IT should, however, be noted that about 70% of the PC patients in our study did not undergo a future cholecystectomy. The major limitation of our patient series is that it represents a retrospective study. In addition, the indications for PC are not strict defined and recommendations for future use should therefore be drawn with caution.

## Conclusion

In conclusion, percutaneous cholecystostomy is a valid alternative for patients with acute cholecystitis Grade II - III. The relief of symptoms is almost instant, the complication rate is minimal and the post-procedure mortality of the same order as that following cholecystectomy in the acute phase.

## Abbreviations

PC: Percutaneous cholecystostomy
LC: Laparoscopic cholecystectomy
CRP: C-reactive protein
PTD: Percutaneous transhepatic drainage
AC: Acute cholecystitis
